# Tight knit under stress: colony resilience to the loss of tandem leaders during relocation in an Indian ant

**DOI:** 10.1098/rsos.150104

**Published:** 2015-09-23

**Authors:** Swetashree Kolay, Sumana Annagiri

**Affiliations:** Behaviour and Ecology Lab, Department of Biological Sciences, Indian Institute of Science Education and Research, Kolkata, Mohanpur, West Bengal 741246, India

**Keywords:** division of labour, network analysis, tandem running, ponerine ant, *Diacamma indicum*

## Abstract

The movement of colonies from one nest to another is a frequent event in the lives of many social insects and is important for their survival and propagation. This goal-oriented task is accomplished by means of tandem running in some ant species, such as *Diacamma indicum*. Tandem leaders are central to this process as they know the location of the new nest and lead colony members to it. Relocations involving targeted removal of leaders were compared with unmanipulated and random member removal relocations. Behavioural observations were integrated with network analysis to examine the differences in the pattern of task organization at the level of individuals and that of the colony. All colonies completed relocation successfully and leaders who substituted the removed tandem leaders conducted the task at a similar rate having redistributed the task in a less skewed manner. In terms of network structure, this resilience was due to significantly higher density and outcloseness indicating increased interaction between substitute leaders. By contrast, leader–follower interactions and random removal networks showed no discernible changes. Similar explorations of other goal-oriented tasks in other societies will possibly unveil new facets in the interplay between individuals that enable the group to respond effectively to stress.

## 1. Introduction

A number of organisms living in diverse environments occupy a nest for at least a part of their life cycle. A substantial amount of time and resources are invested in constructing, maintaining and guarding the nests as these provide the occupants protection from environmental adversities and safe storage space for brood and other resources [1]. Moreover, for social insects like ants, wasps and honeybees, the nest serves as an integral communal platform for rearing their young, coordinating their activities and sharing resources [2–4]. However, at times these nests need to be abandoned and organisms have to relocate to a new shelter due to several factors like physical disturbance to the nest, change in nest microclimate, increased predation and competition [5,6]. Unlike in solitary animals, relocation in social insects is a complex process involving a search for a new nest followed by a coordinated transfer of colony members. This issue has been addressed in detail in honeybees and a few species of wasps and ants [7]. When honeybee colonies need to relocate, scouts use waggle dances to transfer information to other scouts regarding potential new nests, compare between them and, on reaching a consensus, fly as a group to the new nest [8]. In the majority of the ant species, chemical trails are laid along the path to lead colony members from one site to another [5]. Two other modes of recruitment, carrying and tandem running, have also been documented in ants. In the former case, as the name suggests, individuals are lifted and taken from the old nest to the new nest [9]. In the latter, nest-mates (followers) are walked one at a time from one nest to another by individuals (leaders) who have knowledge of the new nest site, while maintaining physical contact throughout the process [10]. In addition to transferring nest-mates, tandem running provides an opportunity for the leader to transfer information about the path to and location of the new nest to other potential leaders [11].

Relocation is a goal-oriented task that initiates with scouts going out in search of a new shelter and terminates when all adults and brood of the colony have been transferred to the new nest. In ants that relocate by tandem running, tandem leaders play a central role in the organization and execution of this task [10]. One of the principal reasons for the ecological success of social insect colonies is the organization of work within the colony [12]. Different tasks are performed simultaneously in the colony by different groups of individuals leading to increased efficiency in task performance [12]. Within a task, there is variability in terms of which colony members perform the task and the amount of work done by each individual [12–14]. Task flexibility can be examined by causing perturbations in the form of random or targeted removal of individuals as well as by increasing or decreasing the total workload. Targeted removal of key individuals can severely affect the organization of work within social insect colonies. When the most active workers involved in a particular task are removed, work rate is lowered and productivity decreases for a period of a few days [15–17]. The tasks performed by removed workers are performed either by the remaining workers who increase their work rate or by task switching of workers involved in other tasks [13].

Colonies of social insects such as ants, wasps and honeybees constitute highly integrated and complex units which exhibit multiple levels of organization. Local interactions between individuals give rise to attributes that are emergent at the colony level and are not apparent by studying pairwise interactions only. Network theory provides a tool to link functionality of the group to behaviour of individuals within the group and to understand both local interactions as well as global colony-level properties [18]. Social network analysis has been used frequently in recent years to study association and interaction patterns and their role in transmission of information and diseases in different groups of social animals [19–21]. A number of studies have looked at how targeted and random removal of individuals, either experimentally or through simulations, affects interaction networks [22–29]. Previous studies have shown that networks tend to become more fragmented and there is a greater increase in network diameter upon targeted removal of individuals than when random individuals are removed [22,27]. Earlier studies have used non-specific interactions like antennation or proximity between individuals to analyse networks in animal societies. By contrast, in this study, we examine the organization of a goal-oriented task using network tools. In addition, we use tandem running, a behaviour that is both directed and has a clear functional connotation, in order to construct the interaction networks. The effects of perturbations in the form of removal of individuals on information flow and organization of colony relocation were examined.

We used the ant *Diacamma indicum* to study task allocation and the effects of perturbations on colony relocation. *Diacamma indicum* is a primitively eusocial queenless ant species belonging to the subfamily Ponerinae recorded from the eastern and southern parts of India and Sri Lanka [30]. Colony sizes are small and range from 20 to 300 monomorphic adults. Each colony has a single reproductive individual known as gamergate. Recent work suggests that tandem running is used to transfer most of the adult workers of the colony during relocation, whereas brood and males are carried [31,32]. Tandem running of adults serves a twofold function in *D. indicum* colonies. Tandem runs are functionally relevant interactions that are used to recruit individuals from the old nest to the new nest. In addition, tandem runs also serve as a means of transfer of information about possible new nesting sites among leaders [11,31]. Since leaders are responsible for performing work related to colony relocation, removing the majority of the leaders would allow us to examine several aspects of relocation in these ants. Targeted removal of tandem leaders would elucidate the importance of tandem running in *D. indicum* and whether tandem running remains the primary mode of relocation despite the loss of leaders. We wanted to examine if secondary leaders emerge and, if so, to analyse the efficiency of these secondary leaders and study work organization among them. We analysed the data from two different approaches: behavioural observations that characterized individual involvement in tandem running, and network analysis that characterized the pattern of interactions at the scale of functional subgroups and the colony itself. Using these two approaches, we examined the impact of leaders' absence on the dynamics of this goal-oriented task.

## 2. Material and methods

Sixteen colonies of *D. indicum* were collected from Mohanpur, West Bengal, India (22°56′ N, 88°31′ E) between September 2013 and May 2014. The colonies were kept in the laboratory in artificial nests inside plastic boxes lined with plaster of Paris and were given ad *libitum* food consisting of ant cake, honey, water and termites [32]. Every ant in the colony was marked with a unique combination of non-toxic enamel paint (Testors, Rockford, IL, USA) on one, two or three locations (first and second thoracic segments and gaster) on its body to enable individual identification. Colonies contained 133.9±37 adults (average ± s.d.), a single gamergate and different stages of brood (pupae, larvae and eggs).

**Figure 1.**
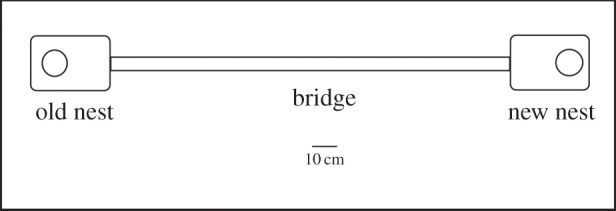
Experimental set-up. This schematic diagram represents the experimental set-up used in the laboratory for conducting relocations. It consisted of two identical nest-boxes connected by a bridge. Within each box, ants were housed in an artificial nest (represented by circles in the figure).

Relocation experiments consisted of allowing colonies to move from their old nest to an identical new nest that was located across a 152-cm-long wooden bridge (figure 1). Colonies were motivated to move by removing the nest cover following standard protocol [32]. Initially ants scout the arena searching for an alternative nesting site, and upon discovering such a site some return to the old nest and initiate tandem running. Eight colonies performed control relocations (CRs) and a set of manipulated relocations termed as leader removal relocation (LRR) in random order on consecutive days. In CR, the colonies were allowed to relocate without any manipulation. In LRR, after initiation of relocation, any leader performing a tandem run within a period of 90 min was removed. The period of 90 min was allocated as removal time as this is close to the average time taken for colonies to search for and relocate to a new nest over similar distances under laboratory conditions (S Kolay 2011, personal observations). This ensured that almost all leaders who would initiate tandem running during a normal relocation were removed. Leaders were picked up with forceps and removed soon after the tandem run started in order to minimize disturbance to the colony. The removed leaders were maintained in a separate box for the remainder of the experiment. After disruption of the tandem run, the followers either returned to the old nest or remained in the location where they had been abandoned. In LRR, 18.6±9.5% of colony members initiated tandem runs and were removed following this protocol. At the end of removal time, removal of tandem leaders was stopped and no further manipulations were done. Any leader who performed tandem runs after this removal period was termed as a substitute leader.

Experimental removal of multiple leaders not only caused physical disturbance but also reduced the effective size of the colony. In order to understand the effect of reduction in colony size on relocation dynamics, additional experiments were conducted in which random colony members were removed. Another eight colonies were subjected to one CR and a manipulated relocation termed as random removal relocation (RRR) performed in random order on consecutive days. CR was performed in a similar manner as in the previous set of experiments. In RRR, soon after initiation of relocation, 20±0.6% of the colony adults who had been randomly pre-selected were removed upon first sighting. Removed individuals were maintained in a separate box till the end of the experiment. After removal of these adults no further manipulations were done.

In all the experiments, data were collected using focal behaviour sampling [33] on tandem runs. Information regarding the identities of the leader and follower, the initiation and termination sites, and the initiation/termination time was recorded for each tandem run and analysed further. For each relocation, relocation time was calculated as the duration between the first tandem run terminating at the new nest and the last such tandem run. The time required to search for the new nest was not included as part of relocation time. Only tandem runs that terminated at the new nest were considered for all statistical analyses. In addition, in the case of LRR, relocation dynamics of only the post-removal period was considered for analysis. Comparisons were carried out using non-parametric two-tailed tests using statistiXL (v. 1.8). Unless mentioned otherwise, mean and standard deviation of various parameters have been presented.

Interaction networks were constructed using adult members of the colony as nodes and tandem runs as edges. As tandem runs were initiated by leaders, these were considered as directed interactions with edges pointing from the leaders to the followers. Some leader and follower pairs were observed to be carrying out multiple tandem runs and, thus, we built weighted networks. Separate tandem running networks were constructed for the control and manipulated relocations with all ants in the colony who had participated in at least one tandem run either as leader or follower. Individuals who reached the new nest following independent exploration were not considered in the network analysis.

In order to understand the dynamics of the interactions, we compared three network-level parameters—density, degree centralization and average closeness. Density is a measure of the proportion of potential edges between nodes that are actually present in a network and can have values ranging from 0 to 1 [34]. High density indicates presence of more edges linking the nodes of a network, while low density indicates fewer links between the nodes. In the case of relocation, followers on reaching a new nest are generally not led on additional tandem runs; thus, we expect density values to be low when a single new nest is provided as is the case in this study. If a follower follows multiple leaders, it will increase the density of the network by creating additional edges between the nodes. Degree centralization gives information regarding the extent of involvement of individuals in the relocation and indicates the prominence of one or a few individuals in the process of relocation [34]. In the case of directed networks, outdegree centralization and indegree centralization can be measured separately. Outdegree centralization is based on the number of edges that originate from each node, i.e. the number of tandem runs performed by the leaders. If a few leaders performed all the tandem runs, the network would have high outdegree centralization. Indegree centralization is calculated from the number of edges directed towards each node, i.e. the number of times each follower is tandem run by leaders. Closeness is a measure of the shortest path linking each pair of nodes within a network, and the average closeness values of all nodes in each network have been presented [34]. High closeness values indicate the presence of direct connections between pairs of individuals in the colony, while low closeness values indicate that colony members were linked to each other indirectly through multiple intermediates. Since the networks are directed, average outcloseness (depending on the number of tandem runs initiated by each individual) and average incloseness (based on the number of times each individual has been tandem run) have been calculated separately.

Following this application of network tools at the colony level, we wanted to examine if the information flow among leaders themselves was different as compared to the interaction between leaders and followers. In order to carry this out, we *post facto* divided all the tandem runs observed during the control, leader removal and RRRs into two categories—tandem runs in which a leader was followed by another leader (LFL) and tandem runs in which a leader was followed by a follower who did not become a leader during the given relocation (FFL). The LFL tandem runs included (i) tandem runs in which leaders recruited followers who later became leaders during the course of the given relocation; and (ii) tandem runs where experienced leaders who had performed tandem runs during the given relocation followed other leaders. Tandem running networks were constructed for LFL and FFL separately for each relocation. The same three network parameters—density, degree centralization and closeness—were calculated for each network. Network analysis was carried out using UCINET 6 for Windows [35]. After obtaining the network parameters for each colony, these were compared using non-parametric tests using statistiXL (v. 1.8).

## 3. Results

All 16 colonies successfully moved to the new nest without splitting into subgroups in control as well as both sets of manipulated relocations. In the LRRs, substitute leaders emerged after initial removal of leaders and they performed tandem runs. The percentage of colony members participating in tandem running to the new nest either as a leader or a follower was higher than individuals that reached the new nest by independent exploration in the control, leader removal and random removal relocations. The percentage of colony members involved in tandem running in LRR (85.3±8.5) was significantly higher than that in CR (77.7±7.6) (Wilcoxon paired sample test, *T*=2.0,*n*=8,*p*=0.02). However, the percentage of the colony involved in tandem running in RRR (84.8±5.1) and CR (74±15.7) was comparable (Wilcoxon paired sample test, *T*=5.0,*n*=8,*p*=0.08). Combined these results illustrate that tandem running is very important in the context of relocation in *D. indicum* as even in the absence of the initial leaders the colonies employed tandem running to relocate.

**Figure 2.**
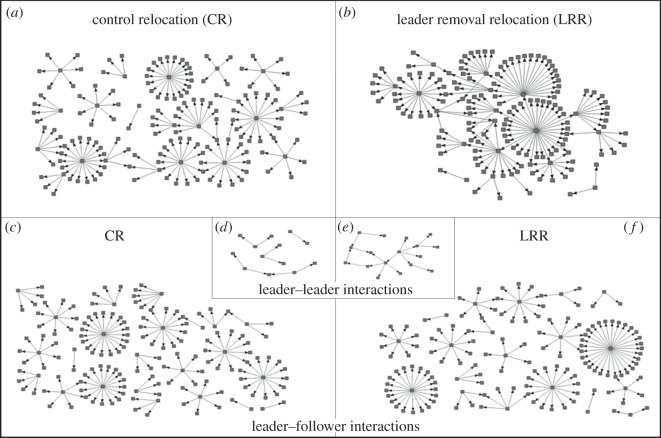
Tandem running networks. Weighted directed interaction networks with individual ants as nodes and tandem runs as directed edges connecting the leader to the follower are presented for a single colony DI-325 which had 192 members. The upper panels represent the interaction networks of the entire colony for (*a*) CR and (*b*) LRR.The lower panels represent the follower following leader (FFL) networks in the (*c*) control and (*f*) LRRs. The insets contain leader following leader (LFL) networks of the (*d*) control and (*e*) LRRs.

The manner in which tandem running was performed by leaders in relocation was analysed using network analysis. The network graph for one colony has been presented in figure 2*a*,*b*. The network sizes in terms of number of nodes were comparable between CR (104.3±27.6) and LRR (95.6±33.6) (Wilcoxon paired sample test, *T*=6.0,*n*=8,*p*=0.1). Starting this analysis at the colony level, we address three parameters—density, degree centralization and closeness. As expected, the density values were very low (see Material and methods) and ranged from 0.006 to 0.028. Nevertheless, density was significantly higher in LRR as compared to CR (table 1). The outdegree centralization and indegree centralization did not change significantly across CR and LRR (table 1). Outcloseness was significantly higher in LRR as compared to CR whereas incloseness was comparable (table 1). At the colony level no significant differences were observed in any of the network parameters between CR and RRR and network sizes were comparable between CR (97±31.7) and RRR (89.8±27.2) (Wilcoxon paired sample test, *T*=11.5,*n*=8,*p*=0.4). Density between CR and RRR was not significantly different (table 2). The outdegree centralization and indegree centralization did not change significantly across CR and RRR (table 2). Outcloseness and incloseness were not different between CR and RRR (table 2).

**Table 1.** Comparison of network parameters of CR and LRR. Average and standard deviation of the various network parameters for control relocation (CR) and leader removal relocation (LRR) at the level of colony, follower following leader (FFL) and leader following leader (LFL) across eight colonies are presented. Critical values and *p*-values obtained by comparing the values of network parameters between the different categories using Wilcoxon paired sample test are also indicated. Comparisons that were significantly different (*p*<0.05) have been indicated in italic.

	density	outcloseness	incloseness	outdegree centralization	indegree centralization
colony					
CR	0.01±0.003	1.05±0.26	1.03±0.26	12.61% ± 6.73%	1.13% ± 0.59%
LRR	0.013±0.006	1.32±0.55	1.23±0.56	16.85% ± 12.55%	0.87% ± 0.7%
	*T*=0.001	*T*=1.5	*T*=4.5	*T*=16.0	*T*=8.0
	*p*=*0*.*03*	*p*=*0*.*02*	*p*=0.1	*p*=0.8	*p*=0.2
FFL					
CR	0.009±0.003	1.03±0.26	1.03±0.26	9.96% ± 5.52%	1.27% ± 0.65%
LRR	0.011±0.006	1.23±0.58	1.22±0.57	15.69% ± 11.85%	0.95% ± 0.78%
	*T*=0.001	*T*=6.5	*T*=7.0	*T*=13.0	*T*=8.0
	*p*=*0*.*03*	*p*=0.1	*p*=0.2	*p*=0.6	*p*=0.2
LFL					
CR	0.06±0.02	7.65±2.69	7.08±2.38	25.58% ± 14.56%	8.3% ± 4.22%
LRR	0.11±0.06	15.58±8.28	13.66±7.71	31.19% ± 17.94%	9.76% ± 11.77%
	*T*=0.001	*T*=1.0	*T*=1.0	*T*=12.0	*T*=14.0
	*p*=*0*.*008*	*p*=*0*.*02*	*p*=*0*.*02*	*p*=0.5	*p*=0.6

**Table 2.** Comparison of network parameters of CR and RRR. Average and standard deviation of the various network parameters for control relocation (CR) and random removal relocation (RRR) at the level of colony, follower following leader (FFL) and leader following leader (LFL) across eight colonies are presented. Critical values and *p*-values obtained by comparing the values of the network parameters between the different categories using Wilcoxon paired sample test are also indicated. Comparisons that were significantly different (*p*<0.05) have been indicated in italic.

	density	outcloseness	incloseness	outdegree centralization	indegree centralization
colony					
CR	0.012±0.004	1.19±0.45	1.17±0.44	16.31% ± 8.46%	1.79% ± 1.14%
RRR	0.014±0.007	1.62±1.08	1.26±0.42	15.79% ± 5.84%	1.67% ± 1.15%
	*T*=3.5	*T* = 9.0	*T* = 12.0	*T* = 16.0	*T* = 14.0
	*p* = 0.09	*p* = 0.3	*p* = 0.5	*p* = 0.8	*p* = 0.6
FFL					
CR	0.01±0.004	1.17±0.43	1.17±0.43	15.41% ± 7.92%	1.73% ± 1.33%
RRR	0.013±0.006	1.25±0.42	1.24±0.42	12.85% ± 4.78%	1.82% ± 1.25%
	*T*=5.5	*T*=10.0	*T*=10.5	*T*=9.0	*T*=15.0
	*p*=0.2	*p*=0.3	*p*=0.3	*p*=0.3	*p*=0.7
LFL					
CR	0.1±0.04	11.52±5.49	11.13±5.24	22.79% ± 13.28%	16.32% ± 12.6%
RRR	0.1±0.07	13.87±10.6	10.72±6.94	33.13% ± 25.45%	9.63% ± 8.77%
	*T* = 17.0	*T*=8.0	*T*=15.0	*T*=12.0	*T*=1.0
	*p* = 0.9	*p* = 0.2	*p* = 0.7	*p* = 0.5	*p*=*0*.*02*

The percentage of substitute leaders who successfully relocated the colony in LRR (12±5.4) was significantly lower than the CR leaders (17.6±5.6) (Wilcoxon paired sample test, *T*=1.0, *n*=8, *p*=0.02). However, the relocation time from the first successful tandem run to the last one was comparable between CR (53.4±36 min) and LRR (55.3±22.8 min) (Wilcoxon paired sample test, *T*=17.0,*n*=8, *p*=0.9), as was the number of tandem runs (CR—103.1±24.7, LRR—98±36.2, Wilcoxon paired sample test, *T*=16.5,*n*=8,*p*=0.9). The rate of colony relocation calculated as number of tandem runs per minute was not significantly different between CR (2.5±1.2) and LRR (2.1±1.3) (Wilcoxon paired sample test, *T*=14.0,*n*=8,*p*=0.6). The percentage of leaders in CR (12.9±3.5) and RRR (15.4±4.4) was comparable (Wilcoxon paired sample test, *T*=10.0,*n*=8,*p*=0.3). In both cases, they took comparable time (CR—76.6±36.4 min, RRR—57.5±19.3 min, Wilcoxon paired sample test, *T*=10.0,*n*=8,*p*=0.3) to perform similar numbers of tandem runs (CR—100.4±38.7, RRR—100.8±27.9, Wilcoxon paired sample test, *T*=17.0,*n*=8,*p*=0.9). The relocation rate was also not different between CR (1.5±0.7) and RRR (1.8±0.4) (Wilcoxon paired sample test, *T*=13.0,*n*=8,*p*=0.5).

**Figure 3.**
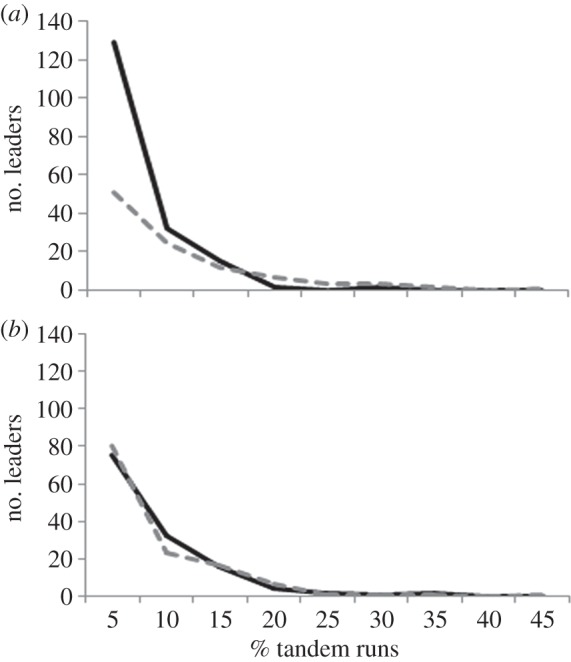
Frequency distribution of tandem running. Frequency distributions of tandem runs performed by leaders in (*a*) CR (black solid line) and LRR (grey dashed line) and (*b*) CR (black solid line) and RRR (grey dashed line) are shown. Percentage of tandem runs is plotted against number of leaders who performed them. Data have been pooled across eight colonies for each type of relocation.

We examined the allocation of tandem runs among leaders by studying the frequency distribution of tandem runs. In all relocations, the contribution of tandem leaders to tandem running was not uniform as some leaders did most of the tandem running while the majority performed only a few, resulting in right skewed frequency distribution of tandem running by the leaders. The distributions were different in CR and LRR (Kolmogorov–Smirnov test, *T*=23.5, d.f.=9, 104, *p*<0.001; figure 3*a*), with a less skewed distribution in LRR indicating that the substitute leaders distributed the task more evenly among themselves. However, these distributions were similar in CR and RRR (Kolmogorov–Smirnov test, *T*=5.6, d.f.=9,131, *p*=1.0; figure 3*b*)

Most of the tandem runs observed were between leaders and followers (FFL) and in the remaining tandem runs leaders were followed by other leaders (LFL) in both CR (FFL—85.5±6.7%, LFL—14.5±6.7%, Wilcoxon paired sample test, *T*=0.0,*n*=8,*p*=0.008) and LRR (FFL—90.1±5.8%, LFL—9.9±5.8%, Wilcoxon paired sample test, *T*=0.0,*n*=8,*p*=0.008). This pattern was echoed in the second set of relocations where FFL tandem runs were significantly higher than LFL tandem runs in CR (FFL—87.5±5.2%, LFL—12.5±5.2%, Wilcoxon paired sample test, *T*=0.0,*n*=8,*p*=0.008) as well as in RRR (FFL—85.7±6.9%, LFL—14.3±6.9%, Wilcoxon paired sample test, *T*=0.0,*n*=8,*p*=0.008).

**Figure 4.**
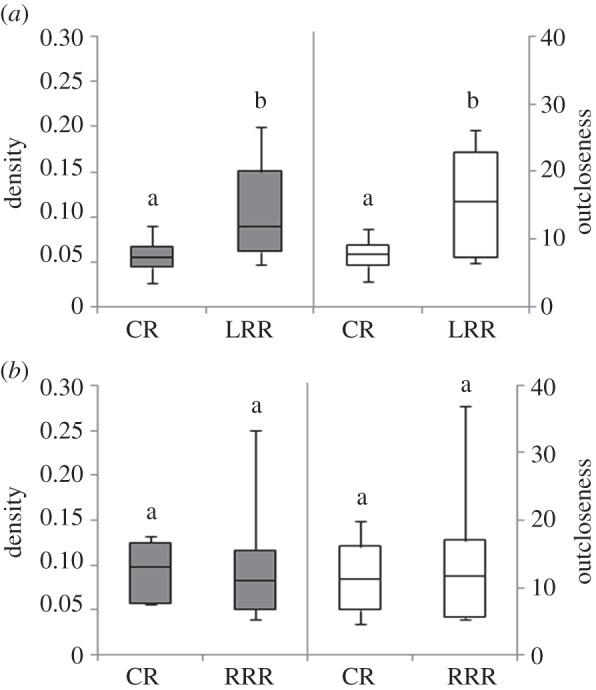
Comparison of network parameters. Density (grey bars, primary *y*-axis) and average outcloseness (white bars, secondary *y*-axis) of leader follow leader (LFL) networks in (*a*) CRs and LRRs and (*b*) CRs and RRRs are presented. Each box represents the interquartile range, the line inside the box represents the median and the whiskers represent the range for data pooled across eight colonies for each set of relocations. Comparisons of parameters were carried out using Wilcoxon paired sample test and boxes carrying different letters are significantly different.

There were differences in the manner in which the leaders interacted with other leaders (LFL) as compared to interactions with followers (FFL) (see the electronic supplementary material, tables S1 and S2). There were also differences in the manner in which substitute leaders interacted with each other. A representative network graph of the FFL and LFL networks of CR and LRR for one colony has been illustrated in figure 2*c*–*f*. On comparing the FFL networks, only density was significantly higher in LRR, whereas the remaining parameters—outcloseness, incloseness, outdegree centralization and indegree centralization—were comparable between CR and LRR (table 1), indicating that number of interactions between leaders and followers increased but the pattern was consistent in the two different circumstances. In the LFL networks, the leaders interacted more among themselves in LRR as indicated by the higher density (figure 4*a* and table 1). Outcloseness and incloseness were higher in the LRR LFL networks, illustrating more direct interactions between leaders resulting in shorter path lengths linking individuals (figure 4*a* and table 1). Outdegree and indegree centralizations of LFL networks were comparable between CR and LRR (table 1). There were no differences in any of the parameters of FFL networks between CR and RRR (table 2). Density, outcloseness, incloseness and outdegree centralization of LFL networks were comparable between CR and RRR; only indegree centralization was significantly lower in RRR LFL (figure 4*b* and table 2). The results from the analysis of FFL and LFL networks illustrate that the pattern of interaction between substitute leaders changed, while leader–follower networks were not impacted.

## 4. Discussion

In this study, we examined the effects of targeted and random removal of workers experimentally on the accomplishment of a goal-oriented task. The removal of workers during relocation would mimic the natural conditions when they get preyed upon or lost en route to the new nest. Tandem running puts both leaders and followers at risk as the speed of a tandem run is expected to be slower in *D. indicum* than if the ants were moving individually along chemical trails as has been seen in other species [11]. However, tandem leaders are especially vulnerable in this respect as they spend extended periods of time outside the nest during relocation. Firstly, they search for alternative nesting sites and, later, transfer nest-mates one at a time from the old nest to the new one. Worker removal was used as a platform to examine two aspects of relocation: firstly, the importance of tandem running as the means of relocation in *D. indicum* and, secondly, flexibility in work organization in the context of colony relocation. By collecting data on the focal behaviour of tandem running, both these aspects were explored at the level of individuals by behavioural analysis and network analysis was used to understand the emergent organization at the level of the colony.

Ants are known to use one or a combination of different methods to relocate. For example, *Camponotus socius* uses chemical trails [36] and adults of *Aphaenogaster senilis* walk to the new nest [37]. *Temnothorax albipennis* is known to use both tandem running and carrying for relocation [38], colony emigration in *Myrmica rubra* involves group recruitment as well as carrying [39], while *Myrmecina nipponica* combines pheromone trails with quorum responses during relocation [40]. Our study reveals that tandem running is the primary means of relocation in *D. indicum* as the majority of the colony was involved in tandem running either as leader or follower during each relocation. Although in our experimental set-up the bridge provided a direct path to the new nest, the majority of the colony members were tandem run to the new nest. Even in cases in which a large fraction of the leaders who performed tandem running was removed, substitute leaders emerged and nearly 85% of colony members were tandem run to the new nest. In fact, the percentage of individuals tandem run was significantly higher in LRR than in control conditions. This indicates the absence of any trail pheromones and we can conclude that tandem running is the primary means used by *D. indicum* colonies to relocate.

The time taken to relocate all colony members to the new nest was comparable between the control and treatment relocations although colony sizes were significantly reduced in all treatment relocations. This was due to the fact that similar numbers of tandem runs were performed in control and treatment relocations. Even though fewer leaders were observed in LRR than in CR, the rate of relocation was comparable between control and LRRs. This indicates that removal of a subset of the workforce did not negatively impact the rate of task performance at least in the short term. This is contrary to what has been seen in other studies where removal of workers involved in a single task affected the rate at which the task is performed for up to several days [15–17]. This successful completion of the task of relocation highlights the resilience of the colony to a substantial loss of the workforce. It is possible that response to high-risk goal-oriented tasks may be adapted differently from continuous long-term tasks. However, a couple of factors may have contributed to the ability of the substitute leaders to maintain the work rate. The colony members having been exposed for a comparatively longer period of time (initial 90 min after removal of nest cover) were probably eager to move to a new shelter. Further, the leaders who emerged after the removal duration had an extended period of time to explore the arena and the new nest enabling them to navigate to the new nest in a better manner. Both these possibilities would have to be explored in future studies.

A closer inspection of division of labour among leaders reveals that all leaders did not perform equally. A few leaders carried out most of the tandem runs while most leaders performed a few, resulting in a skewed pattern of distribution of tandem running. Similar inter-individual variation in interactions and task distribution has been seen in eusocial insects such as honeybees [41] and ants [31,42–44] as well as other social animals [22,25,45]. The distribution of tandem runs among leaders was robust to sudden changes in the colony caused by random removal of individuals. However, this was not the case in LRRs where the distribution of tandem runs was less skewed among leaders. Individual workers in social insect colonies have variable thresholds for responding to an increased demand in task performance [44,46]. There may be a subset of colony members who have a lower threshold for performing tasks related to colony emigration. As a result, these workers have a higher propensity to become leaders than others and they are more likely to initiate relocation and, perhaps, contribute more to the process during emergency. In RRRs, only a few of the leaders were removed; thus, the work distribution was not affected significantly. But in LRRs, when most of the leaders were removed and the colony faced increased stress, we hypothesize that colonies were forced to adopt an ‘all hands on deck’ approach by engagement of as many individuals as possible [47]. We speculate that there is reduced variation in the thresholds for performing tandem runs among substitute leaders resulting in the reduced skew in work distribution observed in LRRs.

Network analysis was used to examine the path of information flow between individuals and to assess the emergent organization of tandem running at the level of the colony during relocation under different circumstances. Density and outcloseness increased upon removal of leaders but these did not change when random individuals were removed. Both behaviour parameters and network parameters were comparable between control and RRRs. Thus, physical disturbance while removing adults does not impact the outcome observed in LRR. In addition, changes in the LRR networks cannot be attributed to change in network size alone since similar numbers of individuals were removed in both sets of manipulated relocations. A mere reduction in number of nodes does not seem to affect work organization within the colony as has been suggested previously [48], whereas targeted removal of leaders during relocation does bring about significant changes in network structure. Previous studies have shown that both targeted as well as random removal of individuals causes network fragmentation and changes the structure of interaction networks in several species of social animals [22–29]. In contrast, we find that relocation networks are robust to loss of random individuals, while targeted removal of leaders impacts the network structure.

A previous study which had looked at relocation of *D. indicum* in natural conditions had found that the leaders interact and lead each other by tandem running to possible new nest sites. This is important to maintain colony cohesion and prevent fragmentation of the colony when multiple nest sites exist [31]. Thus, tandem running is an important means of communication and transfer of information among leaders. In this study where only a single new nest site was provided, we found that the leaders continued to interact among themselves although the number of LFL tandem runs observed was fewer than the number observed in field studies where multiple nest sites are available [31]. This illustrates that communication among leaders is a fundamental aspect of relocation in this species. LFL tandem runs function to initiate other colony members to become tandem leaders as well as to ensure that all leaders recruit followers to the same nest, thereby preventing fragmentation of the colony.

In addition to the results obtained from classical behaviour analysis, network analysis revealed two important features about interaction among individuals during relocation: (i) Leaders interacted differently with other leaders than with followers who did not lead a single tandem run; and (ii) substitute leaders modified interactions among themselves. Interaction networks between leaders and followers were comparable between control and both sets of manipulated relocations. LFL networks did not change when the colony size was reduced or subjected to disturbance of removing individuals. However, when the initial leaders were removed, the interaction pattern among substitute leaders was significantly different. Increased density among substitute leaders indicates a higher level of redundancy in interactions. A single leader being tandem run several times by other leaders, to the same new nest in this case, may seem like a waste of time and energy during emergency. However, this could be essential in transferring information to other leaders about the precise location of the new nest in order to prevent colony fragmentation and, hence, prove to be valuable eventually. Increased closeness values in LFL networks of substitute leaders helped in maintaining several short paths between all pairs of individuals in the colony. This indicates more direct connection between the substitute leaders making them tight knit. Centralization was not significantly different either at the level of the colony or in the LFL networks of CR and LRR, ensuring that multiple leaders continued to be involved in task organization and execution. Although they do not directly function to transfer adult colony members to the new nest, increased instances of leaders following other leaders in LRR ensure smooth and efficient information flow among leaders and presence of several individuals in the colony with knowledge of the new nest site.

In this study, we examined colony relocation, an important task that has direct implications on colony survival and propagation. Targeted removal of colony members in charge of executing this task produced no change in the ability of the colonies to perform the task. However, a combination of behavioural and network analysis revealed that the substitute individuals involved in this process interacted more closely with each other and redistributed the work more evenly as a response. Studying other goal-oriented tasks and how they are organized is essential for us to understand the robustness of societies formed by social insects and other organisms in the face of stress.

## Supplementary Material

Comparisons of network measures It contains 2 tables with extra information regarding network analysis for completeness and transparency of the presentation

## Data accessibility

Data files relevant to this study can be found in the Dryad database (http://dx.doi.org/10.5061/dryad.3nc0g).
