# Supplementary material for: Tight knit under stress: colony resilience to the loss of tandem leaders during relocation in an Indian ant
Source: R Soc Open Sci. 2015 Sep 23;2(9):150104. doi: 10.1098/rsos.150104 (PMC4593672; doi:10.1098/rsos.150104)
Supplement: Comparisons of network measures It contains 2 tables with extra information regarding network analysis for completeness and transparency of the presentation [file rsos150104supp1.pdf]

Tight knit under stress: Colony resilience to the loss of tandem leaders during relocation in an Indian ant.

Swetashree Kolay[1] and Sumana Annagiri[1,*]

[1*]Address for correspondence:

Behaviour & Ecology Lab, Department of Biological Sciences, Indian Institute of Science Education and Research, Kolkata, Mohanpur, West Bengal 741246, India

Email: sumana@iiserkol.ac.in.

Phone: + 91-33-25873017

Fax: + 91-33-25873028

Table S1

	Density	Outcloseness	Incloseness	Outdegree centralization	Indegree centralization
CR FFL vs CR LFL	T=0.001<br>p=**0.01**	T=0.001<br>p=**0.01**	T=0.001<br>p=**0.01**	T=0.001<br>p=**0.01**	T=1.0<br>p=**0.02**
LRR FFL vs LRR LFL	T=0.001<br>P=**0.01**	T=0.001<br>p=**0.01**	T=0.001<br>p=**0.01**	T=6.0<br>p=0.1	T=1.0<br>p=**0.02**

Comparisons of network measures have been carried out between follower following leader (FFL) and leader following leader (LFL) networks for control relocations (CR) and leader removal relocations (LRR) separately (n=8). Values of the respective network parameters have been presented in Table 1. Critical values and p-values obtained based on Wilcoxon Paired Sample test are indicated. Significant values have been indicated in bold. All parameters considered, except outdegree centralization in case of LRR, are significantly higher for LFL networks than the corresponding FFL networks in both CR and LRR. This indicates that leaders interact differently with other leaders as compared to interactions with followers who do not become leaders during the given relocation.

Table S2

	Density	Outcloseness	Incloseness	Outdegree centralization	Indegree centralization
CR FFL vs CR LFL	T=0.001 p=**0.01**	T=0.001 p=**0.01**	T=0.001 p=**0.01**	T=8.0 p=0.2	T=0.001 p=**0.01**
RRR FFL vs RRR LFL	T=0.001 p=**0.01**	T=0.001 p=**0.01**	T=0.001 p=**0.01**	T=0.001 p=**0.01**	T=0.001 p=**0.01**

Comparisons of network measures have been carried out between follower following leader (FFL) and leader following leader (LFL) networks for control relocations (CR) and random removal relocations (RRR) separately (n=8). Values of the respective network parameters have been presented in Table 2. Critical values and p-values obtained by comparing the values of the network parameters between the different categories are indicated. These values are based on Wilcoxon Paired Sample test. Significant values are indicated in bold. All parameters considered, except outdegree centralization in CR, are significantly higher for LFL networks than the corresponding FFL networks in both CR and LRR. This reiterates that leaders interact differently with other leaders as compared to interactions with followers who do not become leaders during the given relocation.
